# Prediction of Protein Cleavage Site with Feature Selection by Random Forest

**DOI:** 10.1371/journal.pone.0045854

**Published:** 2012-09-18

**Authors:** Bi-Qing Li, Yu-Dong Cai, Kai-Yan Feng, Gui-Jun Zhao

**Affiliations:** 1 Institute of Systems Biology, Shanghai University, Shanghai, P. R. China; 2 Key Laboratory of Systems Biology, Shanghai Institutes for Biological Sciences Chinese Academy of Sciences, Shanghai, P. R. China; 3 Shanghai Center for Bioinformation Technology, Shanghai, P. R. China; 4 Beijing Genomics Institute, Shenzhen Beishan Industrial Zone, Shenzhen, People's Republic of China; 5 Children's Hospital of Shanghai, Shanghai Institute of Medical Genetics, Shanghai Jiaotong University School of Medicine, Shanghai, P. R. China; 6 Key Lab of Embryo Molecular Biology, Ministry of Health, China, and Shanghai Lab of Embryo and Reproduction Engineering, Shanghai, P. R. China; University of Alberta, Canada

## Abstract

Proteinases play critical roles in both intra and extracellular processes by binding and cleaving their protein substrates. The cleavage can either be non-specific as part of degradation during protein catabolism or highly specific as part of proteolytic cascades and signal transduction events. Identification of these targets is extremely challenging. Current computational approaches for predicting cleavage sites are very limited since they mainly represent the amino acid sequences as patterns or frequency matrices. In this work, we developed a novel predictor based on Random Forest algorithm (RF) using maximum relevance minimum redundancy (mRMR) method followed by incremental feature selection (IFS). The features of physicochemical/biochemical properties, sequence conservation, residual disorder, amino acid occurrence frequency, secondary structure and solvent accessibility were utilized to represent the peptides concerned. Here, we compared existing prediction tools which are available for predicting possible cleavage sites in candidate substrates with ours. It is shown that our method makes much more reliable predictions in terms of the overall prediction accuracy. In addition, this predictor allows the use of a wide range of proteinases.

## Introduction

Proteinases are enzymes that play important roles in both cellular and extracellular processes by binding and cleaving their protein substrates. They account for ∼2% of all gene products and are of great importance in medicine and biotechnology because their functions can easily be modulated by small peptide inhibitors or chemical compounds [1]. The cleavage often activates, inactivates, or modifies the substrate, and thus controls a diverse range of biological processes, including the removal of abnormal proteins, stress response, cell cycle control, cell differentiation, metabolic adaptation and immune response of host [2]. Inappropriate proteolytic activity can lead to destructive consequences and results in numerous human diseases [1]. Therefore, a large number of studies have focused on identifying the target substrates and inhibitors of proteases in various disease states, with the ultimate goal of designing appropriate therapy [3], [4].

Conventional approaches of identifying proteinase specificity by searching the enzyme against a library of peptides are time-consuming and labor-intensive. Other techniques are based on genetic or proteome analytical approaches [5]. Computational methods can be used to predict the possible substrates of the proteinases and the probable location of the cleavage sites based on the existing data. Some tools have already been developed for this purpose. At present three proteasome cleavage prediction methods are publicly available on the Internet: PAProC (Prediction Algorithm for Proteasomal Cleavages) (www.paproc.de) which is a prediction tool for cleavages by human and yeast proteasomes, based on experimental cleavage data [6], MAPPP (www.mpiib-berlin.mpg.de/MAPPP/) developed at the Max-Planck Institute in Berlin [7], [8] and NetChop (www.cbs.dtu.dk/services/NetChop/) developed at the Center for Biological Sequence analysis at the Technical University of Denmark which applied artificial neural network to the predictions of cleavage sites of the human proteasome [2], [9].

Besides the three methods mentioned above, most other tools are proteinase specific, such as GPS-CCD for the prediction of calpain cleavage sites [10], GraBCas for the prediction of sites cleaved by granzyme B and caspases [11] and CaSPredictor for caspase substrate prediction [12]. Therefore, these tools are limited in applicability. In this work, we developed a novel predictor based on Random Forest algorithm (RF) using maximum relevance minimum redundancy (mRMR) method followed by incremental feature selection (IFS). We incorporated features of physicochemical/biochemical properties, sequence conservation, residual disorder, amino acid occurrence frequency, secondary structure and the solvent accessibility to code a residue. Five-fold cross validation method was used to evaluate the performance of the classifier. From a total of 704 features, 65 features, regarded as the optimal features, were selected by mRMR and incremental feature selection. The predictor achieved an overall accuracy of 85.45% and MCC of 0.5908 on an independent dataset. Feature analysis showed that all the features contributed to the identification of protein cleavage sites, especially the PSSM score, amino acid factors and amino acid frequency. It was also shown via the site-specific feature analysis that the features of cleavages sites themselves might contribute to the cleavage site determination. It is anticipated that our prediction method may become a useful tool in identifying the protein cleavages sites and that the feature analysis described in this paper may provide some useful insights for in-depth investigations into the mechanism of protein cleavage.

## Materials and Methods

### Dataset

First, we downloaded the protein sequences containing cleavage sites from the UniProt database (version 2011_12) [13]. After removing those without experimentally verified protein cleavage sites, 491 protein sequences were left, containing 846 protein cleavage sites. Finally, we removed proteins whose disorder feature could not be calculated, after which 459 proteins with 712 cleavage sites were left.

The protein cleavages sites annotated in Uniprot are two consecutive amino acids. We selected four different window sizes including 16, 18, 20 and 22 when extracting positive and negatives samples. Then we extracted segments centered on the cleavage sites, with 7, 8, 9 and 10 residues upstream and downstream of the cleavage site, respectively. For a segment with length less than a window size, we complement it with “X”. We regarded a protein segment centered on the cleavage sites as positive data. We also extracted negative data centered on non-cleavage sites. We randomly selected 371 proteins as training dataset and the rest 88 proteins as testing dataset. In the training dataset, there were a total of 578 positive samples and 1734 negatives samples which were randomly selected. In the testing dataset, there were a total of 134 positive samples and 402 randomly selected negative samples. The positions of protein cleavage sites in the protein sequences concerned and their accession numbers were provided in Supporting Information S1.

### Feature Construction

#### The features of PSSM conservation scores

Evolutionary conservation plays important roles in biological analysis. A more conserved residue within a protein sequence may indicate that it is more important for the protein function and thus under stronger selective pressure. We used Position Specific Scoring Matrix (PSSM) generated by Position Specific Iterative BLAST (PSI BLAST) [14] against UniRef100 database (Release: 15.10, 03-Nov-2009) through 3 iterations with 0.0001 as the E-value cutoff to measure the conservation status for a specific residue. A 20-dimensional vector was used to denote the probabilities of conservation against mutations to 20 different amino acids for a specific residue. For a given sequence, all such 20-dimentional vectors for all residues composed the position specific scoring matrix (PSSM). In this study, we used PSSM conservation score to quantify the conservation status of each amino acid in a protein sequence.

#### The features of amino acid factors

Since each of the 20 amino acids has specific but different properties, the composition of these properties of different residues within a protein can influence the specificity and diversity of the protein structure and function. AAIndex [15] is a database containing various physicochemical and biochemical properties of amino acids. Atchley et al. [16] performed multivariate statistical analyses on AAIndex and transformed AAIndex to five multidimensional and highly interpretable numeric patterns of attribute covariation reflecting polarity, secondary structure, molecular volume, codon diversity, and electrostatic charge. We used these five numerical pattern scores (denoted as “amino acid factors” (AAFactor)) to represent the respective properties of each amino acid in a given protein.

#### The features of disorder score

Protein segments lacking fixed three-dimensional structures under physiological conditions play important roles in biological functions [17], [18]. The disordered regions of proteins allow for more modification sites and interaction partners and always contain post translational modification (PTM) sites, sorting signals, and protein ligands. Therefore they are quite important for protein structure and function [17], [19], [20]. In this study, VSL2 [21], which can accurately predict both long and short disordered regions in proteins, was used to calculate disorder score that denotes the disorder status of each amino acid in a given protein sequence.

#### The features of secondary structure and solvent accessibility

The structure of a protein plays an important role for its function. Also, the post-translational modification of specific residues may be affected by their solvent accessibility. In view of this, here we also used the structure features including the secondary structure and the solvent accessibility to encode the peptides. These features were predicted by the solvent accessibility and secondary structure predictors SSpro4 [22], which can be used to predict the secondary structural property of each of the constituent amino acids as ‘helix’, ‘strand’, or ‘other’, encoded with “100”, “010” and “001” respectively. It can also be used to predict the solvent accessibility of each amino acid as ‘buried’ or ‘exposed’, encoded with “10” and “01” respectively.

#### The features of amino acid frequency around protein cleavage sites

We calculated the occurrence frequency for each of the 20 native amino acids as well as the complemented element “X” for the 578 protein cleavage sites derived from the 371 proteins in the training dataset (see Supporting Information S2) and represented it with WebLogo (http://weblogo.berkeley.edu/) [23].

#### The feature space

For each residue of a protein segment, we incorporated 32 features, including 20 features of PSSM conservation score, 1 disorder feature, 5 features of AAFactor, 2 features of solvent accessibility, 3 features of secondary structure and 1 feature of amino acid frequency. Thus, a segment with size-16 sliding window would contain 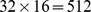 features; with size-18 sliding window, 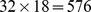; with size-20 sliding window, 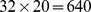
and with size-22 sliding window, 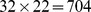. For segments complemented with “X” residues, all features of these “X” residues are denoted as 0.

#### mRMR method

We used Maximum Relevance Minimum Redundancy (mRMR) method to rank the importance of the features [24]. mRMR method could rank features based on both their relevance to the target and the redundancy among the features. A smaller index of a feature denotes that it has a better trade-off between maximum relevance to target and minimum redundancy.

Both relevance and redundancy were quantified by mutual information (MI), which estimates how much one vector is related to another. The MI equation was defined as below:
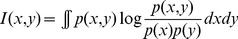
(1)In equation (1), 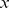, 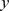
are vectors, 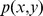
is their joint probabilistic density, and 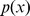
and 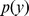
are the marginal probabilistic densities.


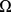
was used to denote the whole feature set. 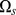
was used to denote the already-selected feature set containing *m* features and 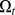
was used to denote the to-be-selected feature set containing *n* features. The relevance 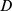 between the feature 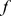 in 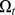 and the target 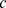 can be calculated by:
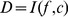
(2)


The redundancy
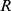
between the feature 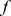
in 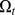 and all the features in 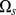 can be calculated by:
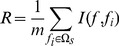
(3)


To get the feature 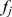 in 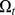
with maximum relevance and minimum redundancy, the mRMR function combines equation (2) and equation (3) and is defined as below:
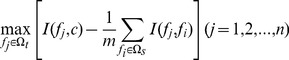
(4)


The mRMR feature evaluation would continue N rounds when given a feature set with N (N = m+n) features. After the calculation, we get a feature set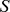:
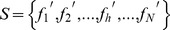
(5)


In this feature set 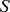, the index h of each feature indicates at which round that the feature is selected. The smaller the index h is, the earlier the feature satisfied equation (4) and the better the feature is.

#### Random Forest

The Random forest (RF) approach is a popular machine-learning algorithm that has been recently successfully used in dealing with various biological prediction problems [25], [26]. Developed by Loe Breiman [27], RF is an ensemble predictor that consists of multiple decision trees. To classify a new queried sample with an input vector, the input vector is predicted by each decision tree in the forest. Each tree provides a predicted class. And the class with the most votes will be output as the predicted class of the random forest. Each tree is constructed using the following procedure:

1. Suppose the number of training cases is N, take N samples at random, but with replacement from the original data, which will be the training set for growing the tree.
2. If there are M input variables, choose a number *m* which ought to be much less than M. At each node, *m* variables are selected randomly out of the M variables and the most optimized split on these *m* variables is employed to split the node. The value of *m* does not change during the growth of the forest.
3. Each tree is fully grown and not pruned.

In this study, we employed Random Forest developed in Weka 3.6.4 [28], which implements the algorithm described above. Notably, it was run with default parameters.

#### Five-fold Cross-Validation Method

Five-fold cross-validation was often used to evaluate the performance of a classifier [29]. In five-fold cross-validation the data are first divided equally into five folds. Subsequently, each fold of data is in turn used as the test data and the remaining 4-folds of data as the training data. Thus, each data is tested exactly once. To evaluate the performance of the predictor, the prediction accuracy, specificity, sensitivity and MCC (Matthews's correlation coefficient) were calculated below:
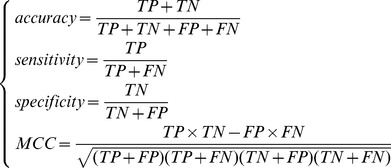
(6)where TP denotes true positive. TN denotes true negative. FP denotes false positive and FN denotes false negative.

#### Incremental Feature Selection (IFS)

Based on the features ranked by mRMR, we used Incremental Feature Selection (IFS) [26], [30] to determine the optimal number of features. During IFS procedure, features in the ranked feature set are added one by one from higher to lower rank. A new feature set is composed when one feature is added. Thus N feature sets will be composed when N ranked features are given. The i-th feature set is:
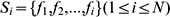
(7)


For each of the N feature sets, a random forest is constructed and tested using five-fold cross-validation test. With N prediction accuracies, sensitivities, specificities and MCCs calculated, an IFS table is obtained with one column being the index i and the other columns being the prediction accuracies, sensitivities, specificities and MCCs. We then can get the optimal feature set (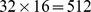), using the predictor that achieves the best prediction performance.

## Results and Discussion

### The mRMR result

After running the mRMR software, we obtained two tables (Supporting Information S3): one is called MaxRel feature table that ranks the features according to their relevance to the class of samples; and the other is called mRMR feature table that lists the ranked features by the maximum relevance and minimum redundancy to the class of samples. In the mRMR feature table, a feature with a smaller index implies that it is more important for protein cleavage site prediction. Such list of ranked feature was to be used in the following IFS procedure for the optimal feature set selection.

### IFS result

**Figure 1:**
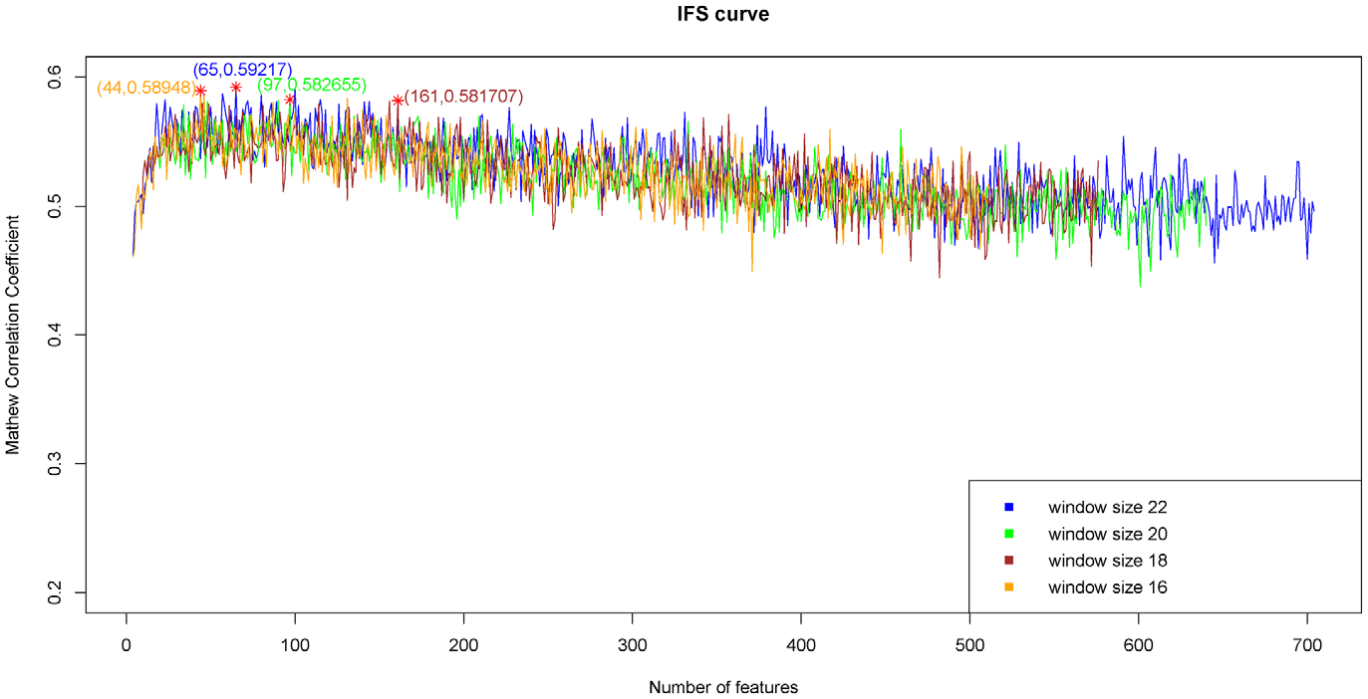
Plot to show the values of MCC against different number of features used based on the data in Supporting Information S4. When the 65 features were used, a peak of MCC was obtained. These 65 features were considered as the optimal feature set for our classifier.

For window size 22, by adding the ranked features one by one, we built 704 individual predictors for the 704 sub-feature sets to predict the protein cleavage sites. We then tested the prediction performance for each of the 704 predictors and obtained the IFS results (Supporting Information S4). Shown in Fig. 1 is the IFS curve plotted based on the data of Supporting Information S4. The same calculations were also carried out for the size-16, size-18 and size-20 windows, and the corresponding results were also plotted in Fig. 1, from which we can see that the predictor based on the size-22 window outperformed the other three, and that the maximal MCC was 0.5922 when 65 features as given in Supporting Information S5 were used. Such 65 features were regarded as the optimal feature set for our classifier. Based on these 65 features, the prediction sensitivity, specificity and accuracy were 61.07%, 93.66%, and 85.51%, respectively (Table 1). From Table 1, we can see that for the same training data set, the performance of window size 22 is better than the other three. For the testing data set, the performance of window size 22 is better than that of window size 16, and comparable with those of window size 18 and 20, though a bit worse. Normally an optimal model is chosen based on the results of cross validation of training set. Therefore, size 22 is chosen as the optimal window size and all the further analyses will be based on the 65 optimal features obtained from window size-22.

**Table 1:** The predicted results obtained with different window size.

Window size	Dataset	Sn	Sp	Ac	MCC
16	train	61.42%	93.37%	85.38%	0.5895
	test	61.19%	92.79%	84.89%	0.5768
18	train	57.96%	94.41%	85.29%	0.5817
	test	61.19%	95.27%	86.75%	0.6253
20	train	59.34%	93.89%	85.25%	0.5827
	test	59.70%	95.77%	86.75%	0.6239
22	train	61.07%	93.66%	85.51%	0.5922
	test	61.19%	93.53%	85.45%	0.5908

Sn: sensitivity.

Sp: specificity.

Ac: accuracy.

MCC: Matthews's correlation coefficient.

### Analysis of the optimal feature set

**Figure 2:**
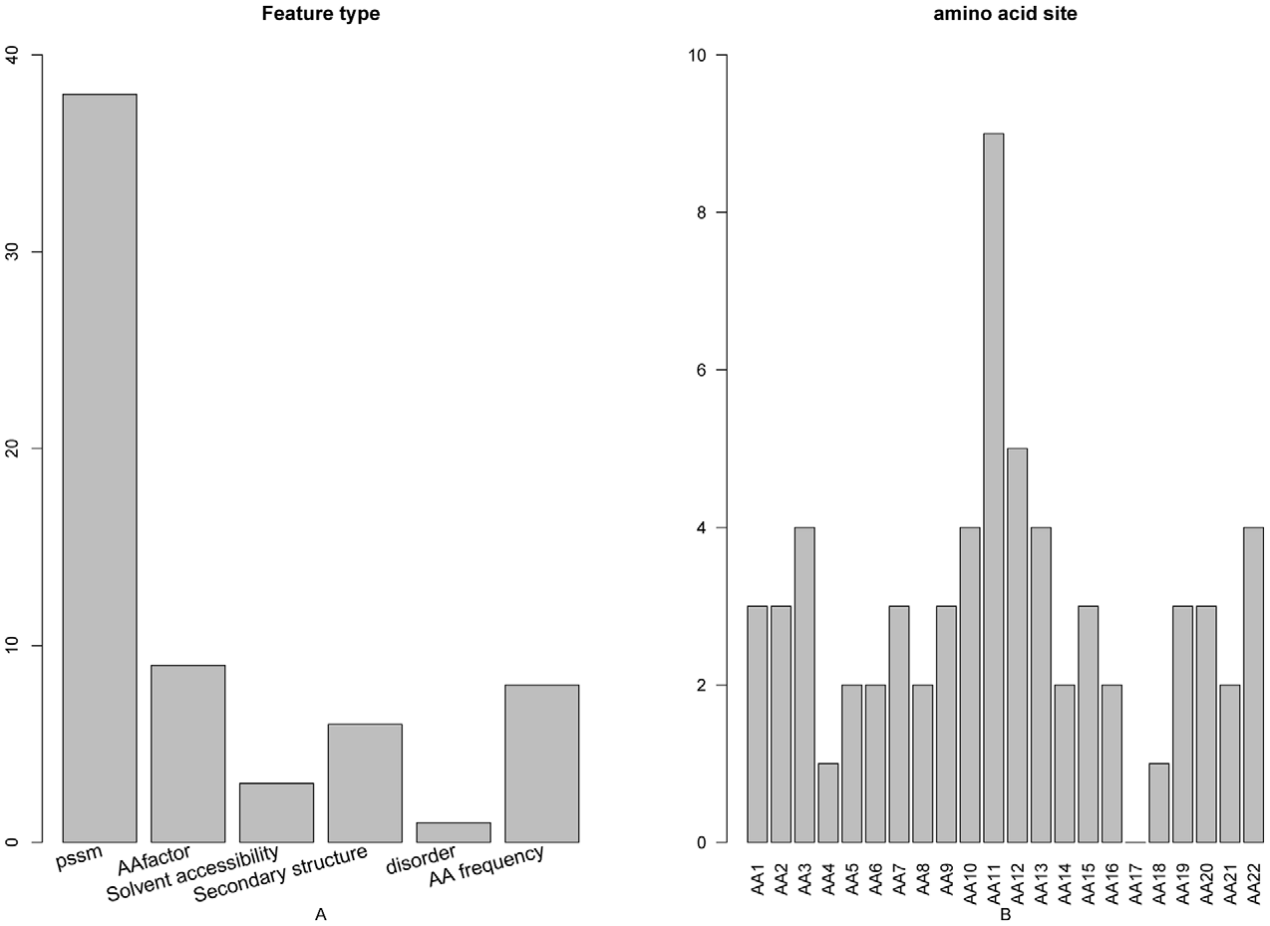
Bar plots to show the feature distribution for the 65 optimal features and the corresponding site distribution. See the section of “Analysis of the optimal feature set” for further explanation.

The distribution of the number of each type of features in the final optimal feature set was investigated and shown in Fig. 2A. As we can see from the figure, of the 65 optimal features, 38 belong to the PSSM conservation score, 9 to the amino acid factor, 6 to the secondary structure, 3 to the solvent accessibility, 8 to the amino acid occurrence frequency, and 1 to the disorder, suggesting that all the six types of features contributed to the prediction of protein cleavage sites. The site-specific distribution of the optimal feature set (Fig. 2B) revealed that site 11 and site 12 played the most important role in the determination of cleavage sites.

### PSSM conservation score feature analysis

**Figure 3:**
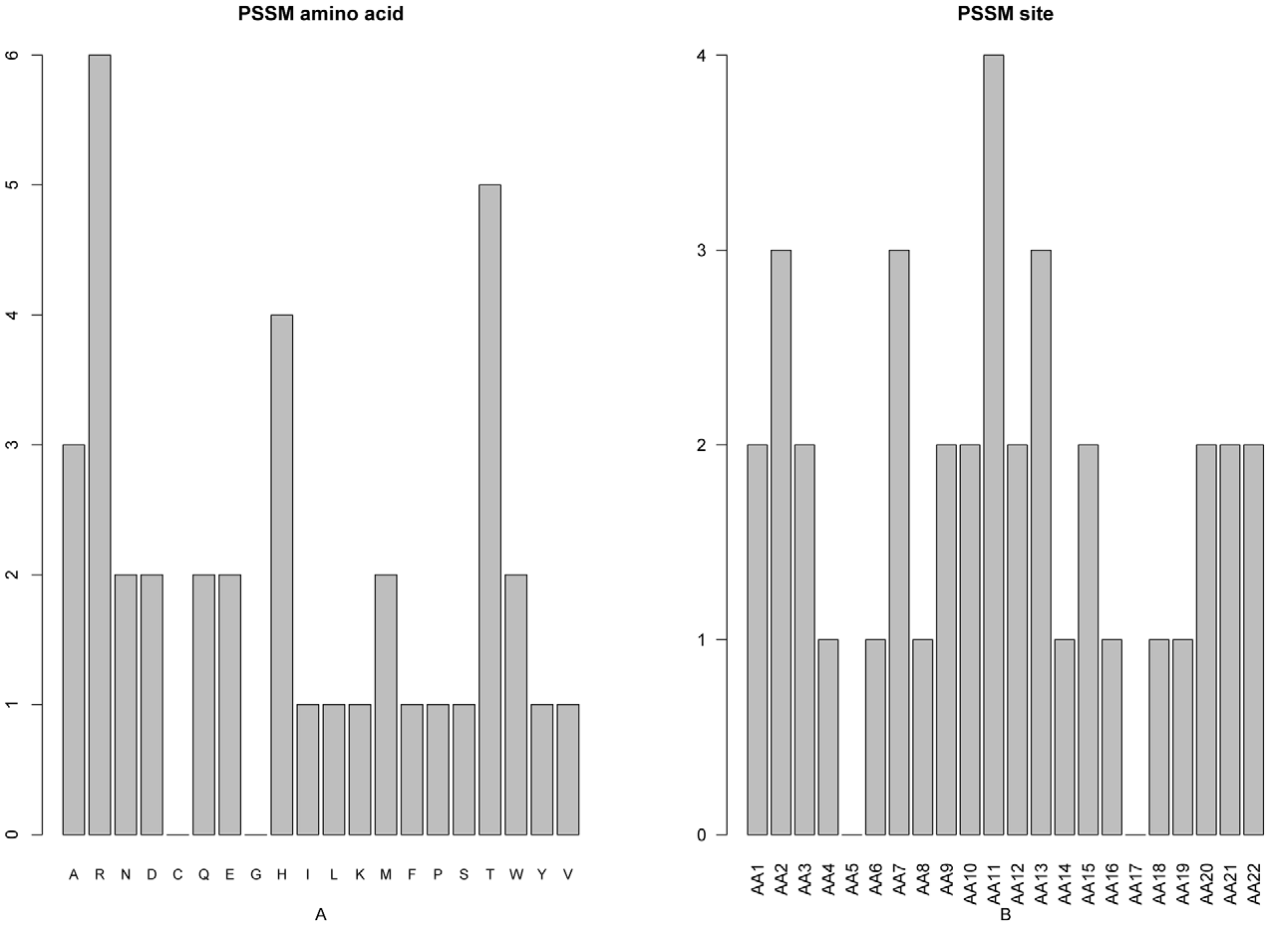
Bar plots to show the distribution in the optimal feature set for the PSSM score and the corresponding specific site score. See the section of “PSSM conservation score feature analysis” for further explanation.

As mentioned above, there were 38 PSSM conservation features, which account for the greatest proportion of the optimal 65 features. We investigated the number of each kind of amino acids for the PSSM features (Fig. 3A) and found that the conservation against mutations to the 20 amino acids contribute differently to the protein cleavage site prediction. Mutations to amino acid Arginine (R), Threonine (T) and Histidine (H) contribute most to the protein cleavage site determination. It has been reported that all 130 furin cleavage sites collected from the published literatures have a positively charged R residue. Furthermore, a mutation of this R diminished the detectable furin cleavage indicating that R is required at furin cleavage site [31], [32], [33], [34]. We also investigated the number of PSSM features at each site (Fig. 3B). The conservation status of site 11 contributed most to protein cleavage site prediction, successively followed by site 2,7, 13 and so forth, as shown in Fig. 3B. In addition, the features within the top 10 features in the final optimal feature list contain four PSSM conservation features: the conservation status against residue Valine (V) at site 11 (index 5, “AA11_pssm_20”), the conservation status against residue Aspartic acid (D) at site 10 (index 6, “AA10_pssm_4”), the conservation status against residue Alanine (A) at site 15 (index 7, “AA15_pssm_1”) and the conservation status against residue Arginine (R) at site 7 (index 8, “AA7_pssm_2”).

### Amino acid factor analysis

**Figure 4:**
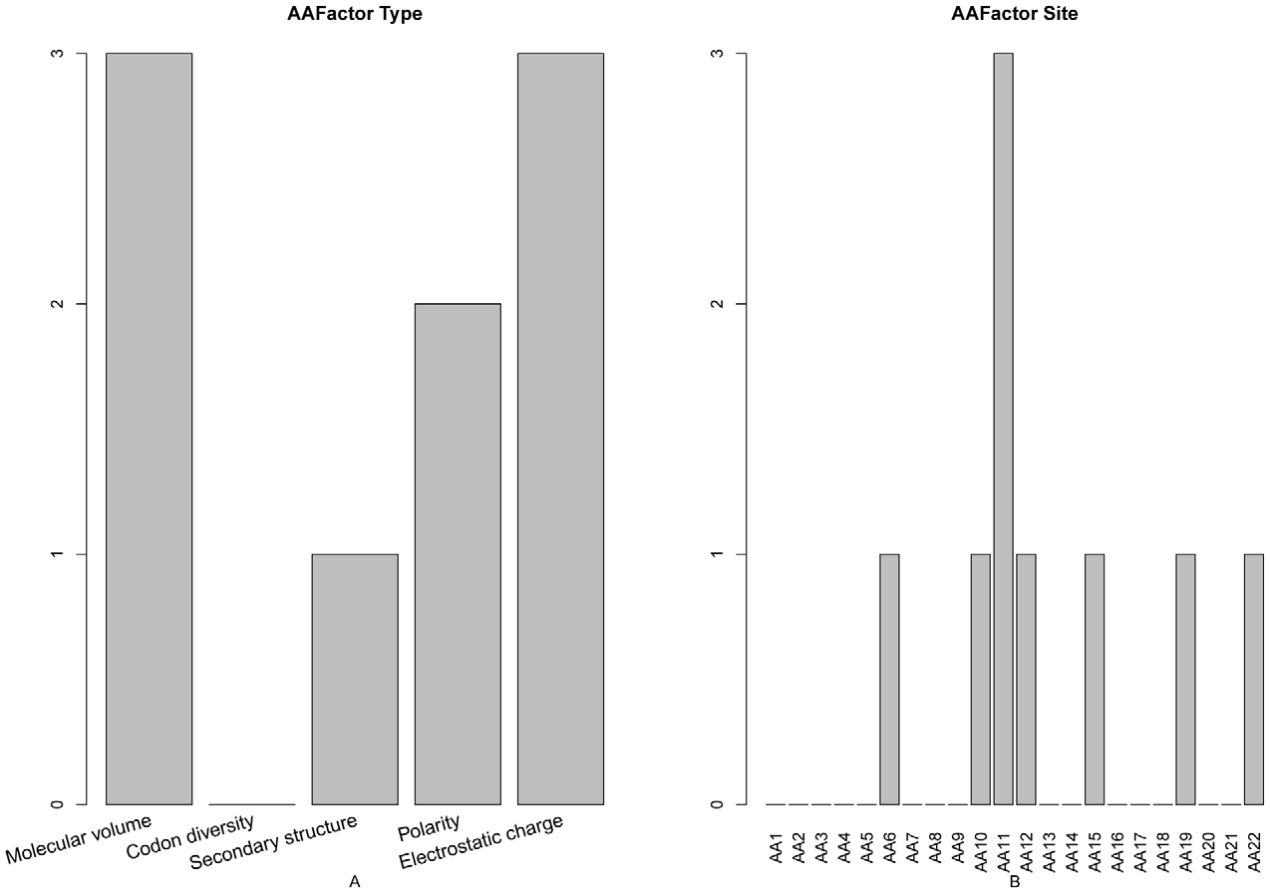
Bar plots to show the distribution in the optimal feature set for the amino acid factor features and the corresponding specific site score. See the section of “Amino acid factor analysis” for further explanation.

The number of each type of amino acid factor features (Fig. 4A) and the number of amino acid factor features at each site (Fig. 4B) were analyzed. It was found that the molecular volume and electrostatic charge was the most important feature, and polarity was the second most important feature to the protein cleavage site prediction. We have mentioned above that all 130 furin cleavage sites compiled from published papers have a positively charged R residue [31], [32], [33], [34], which suggested that electrostatic charge was important for protein cleavage. In Fig. 4B, residue at site 11 had the most effect on the protein cleavage site prediction. The polarity feature of site 22 has an index of 9 in our optimal feature set, indicating that it is one of the most important features for the cleavage site prediction. The molecular volume of site 11 has an index of 10 in our optimal feature set, implying that it also had a crucial role for the cleavage site prediction.

### Disorder analysis

Within the final optimal feature set, only one disorder feature was selected, which suggested that the disorder status may not be important for the prediction of protein cleavage site. This finding was consistent with that proteases universally recognize beta strands in their active sites [35], [36] and strands are more likely to be observed in the vicinity of the cleaved site [37].

### Solvent accessibility features analysis

We investigated the 3 solvent accessibility features in the optimal feature set. All the 3 solvent accessibility features were buried, indicating that the protein cleavage site was skewed toward inaccessible areas. The crystal structures solved showed that the angiotensin cleavage site is inaccessibly buried in its amino-terminal tail [38]. Besides, the Tyr-Met cleavage site of von Willebrand factor (VWF) is buried in a hydrophobic pocket [39]. In addition, it has been shown that the initial and major protease cleavage sites are buried deep within the capsid of human rhinovirus [40]. Furthermore, it was reported that transmembrane domain of the substrate protein must be at least partially unwound and exposed to the aqueous environment before or during the cleavage to allow water access to the cleavage site buried in the membrane [41]. Analysis on site-specific distribution of solvent accessibility features showed that features at site 3, 5, and 19 contribute more to the protein cleavage site determination.

### Secondary structure features analysis

Analysis on the feature and site-specific distribution of the secondary structure in the optimal feature set revealed that secondary structure features of strand and other can affect the protein cleavage site determination and secondary structure features of strand have more impact. The secondary structure features at site 1, 5, 10, 12, 20 and 22 contribute more to the protein cleavage site determination. It has been shown that proteases universally recognize beta strands in their active sites [35], [36]. Liwen You had found that strands are more likely to be observed in the vicinity of the cleaved site [37].

### Amino acid frequency features analysis

**Figure 5:**
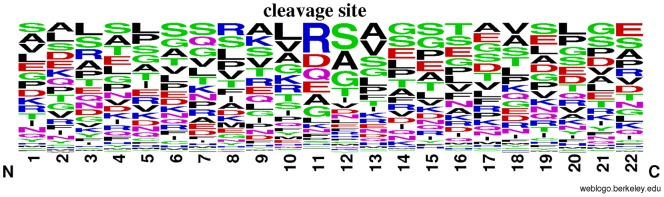
A sequence logo illustration generated by WebLog to show the occurrence frequency of amino acid surrounding the protein cleavage sites. where N and C represent the N- and C-terminus of the 22-residue peptide, respectively, with the cleavage sites occurring at the site 11 and site 12.

The amino acid occurrence frequencies surrounding the protein cleavage sites were represented with WebLogo (http://weblogo.berkeley.edu/) as shown in Fig. 5, from which we can see that there was a high tendency to be Arginine (R) and Aspartic acid (D) at site 11 and Serine (S), Alanine (A) and Glycine (G) at site 12. It has been shown that there is a R at site 11 in all the 130 furin cleavage sites compiled from published papers [31], [32], [33], [34]. Besides, there is a D at site 11 in all the human caspase-3 cleavage sites and a high tendency to be G and S at site 12 [3]. For human calpain-2, it is more likely to be S at site 12 of their cleavage sites [3]. In addition, analysis of 234 general proprotein convertase cleavage site showed that it preferred to be R at site 11 and S at site 12 [42]. Furthermore, it has been reported that there is a high tendency to be R at site 11 and A at site 12 of chloroplast transit peptides cleavage sites [43]. There were a total of 8 amino acid frequency features from site 3, 8, 9, 11–14 and 16 in the optimal feature set. The AA frequency at site 1 had the index of 1 in the optimal feature set, and the AA frequency at site 12 and site 13 had the index of 3 and 4, respectively (see Supporting Information S5). Furthermore, these eight AA frequency features were ranked ahead of the other counterparts in the MaxRel feature table (Supporting Information S3), suggesting that such eight features are more relevant to protein cleavage site prediction. Overall speaking, the AA frequency is a special feature that would play a pivotal role for protein cleavage site prediction.

### Comparisons with other methods

We compared the prediction performance of our method with the three proteasome cleavage prediction methods PAProC [6], MAPPP [7], [8] and NetChop [2], [9] on the same independent testing dataset. For PAProC, there were three different types of human proteasome, type I, type II and type III. Thus, this tool was implemented for three times with different settings corresponding to each human proteasome. We set different threshold for each run and the prediction result with the maximum MCC was regarded as the optimal prediction. As shown in Table 2, overall, the PAProC obtained rather high specificity that is near to 1 (type I) or even 1 (type II and type III), but the sensitivity was extremely low, and the final MCC was poor. In contrast to PAProC, MAPPP had high sensitivity but quite low specificity and MCC was poor (Table 2). For Netchop, there were two different prediction methods, C term 3.0 and 20S 3.0. The best prediction result for each method were listed in Table 2. We can see that like MAPPP, the C term 3.0 method had a high sensitivity, low specificity and poor MCC. While the 20S method achieved a relatively balanced sensitivity and specificity, but the overall MCC was still poor. In summary, our method achieved the highest MCC and outperformed these three online methods on an independent testing dataset.

**Table 2:** Comparison with three proteasome cleavage prediction methods.

method	Sn	Sp	Ac	MCC
ours	61.19%	93.53%	85.45%	0.5908
PAProC(human proteasome type I)	5.22%	98.01%	74.81%	0.0849
PAProC(human proteasome type II)	0.75%	100%	75.19%	0.0749
PAProC(human proteasome type III)	1.49%	100%	75.37%	0.1060
MAPPP	94.03%	16.17%	35.63%	0.1288
NetChop(C term 3.0)	97.01%	9.45%	31.34%	0.1042
NetChop(20S 3.0)	67.91%	40.55%	47.39%	0.0753

Sn: sensitivity.

Sp: specificity.

Ac: accuracy.

MCC: Matthews's correlation coefficient.

### Directions for experimental validation

It is worthwhile to point out that the selected features at different sites may provide useful clues for experimental scientists to find or validate new determinants for protein cleavage. For example, it was found in this study that mutations to amino acid Arginine (R) and electrostatic charge contribute most to the protein cleavage site determination, which has been explicitly validated by previous studies [31], [32], [33], [34]. In addition, it was found through our study that protein cleavage site was skewed toward inaccessible areas, which was consistent with the observations reported in [38], [39], [40], [41]. It was revealed in our studies that the secondary structure of strand played an crucial role in the prediction of protein cleavage site, which was supported by the founding that proteases universally recognize beta strands in their active sites [35], [36], [37]. Moreover, we found there was a high tendency to be Arginine (R) and Aspartic acid (D) at site 11 and Serine (S), Alanine (A) and Glycine (G) at site 12 of protein cleavage sites, which were consistent with numerous previous studies [3], [31], [32], [33], [34], [42], [43]. Accordingly, the remaining features in the optimal feature set are worthy of validation by experiments and further investigations.

## Conclusion

In this study, we developed a new method for predicting and analyzing protein cleavage sites. Our method considered not only the sequence conservation information but also the physicochemical features, solvent accessibility, secondary structure and residue disorder status of protein cleavage sites. Besides, we also took the amino acid occurrence frequency around the protein cleavage sites into consideration. By means of the feature selection algorithm, an optimal set of 65 features were selected; these features were regarded as the ones that contributed significantly to the prediction of protein cleavage. With the 65 optimal features thus selected, our approach achieved an overall accuracy of 85.51% and MCC of 0.5922, and outperformed the existing three web tools when tested by an independent dataset. These selected features may shed some light into in-depth understanding of the mechanism of protein cleavage, providing guidelines for experimental validation.

## Supporting Information

Supporting Information S1 **Positions of the protein cleavage sites in the protein sequences and the accession numbers of the corresponding proteins.** This file contains two sheets. The first one shows the positive and negative samples in training dataset and the second one shows the positive and negative samples in testing dataset.
(XLSX)

Supporting Information S2 **The amino acid frequency around the protein cleavage sites.**
(XLSX)

Supporting Information S3 **This file contains two sheets.** The first one shows the MaxRel feature table ranked according to the relevance between the features and the class of the samples. The second one shows the mRMR feature table ranked according to the redundancy and relevance to the features of the samples.
(XLSX)

Supporting Information S4 **The sensitivity (Sn), specificity (Sp), accuracy (Ac), Matthews's correlation coefficient (MCC) generated by each run of the IFS.**
(XLSX)

Supporting Information S5 **The optimal 65 features selected by IFS.**
(XLSX)
